# Tightly Coupled LEO SOP/INS Integrated Navigation Using an Adaptive Unscented Kalman Filter Framework

**DOI:** 10.3390/s26175593

**Published:** 2026-09-03

**Authors:** Yuan Zhao, Jinghan Feng, Yu Jiang, Zhe Fan, Bing Xie, Chengkai Tang, Yangyang Liu

**Affiliations:** 1 School of Electronics and Information, Northwestern Polytechnical University, Xi’an 710072, China; zhaoyuan90@mail.nwpu.edu.cn (Y.Z.);; 2 School of Information Engineering, Xi’an Mingde Institute of Technology, Xi’an 710124, China; 3 Department of Network Security, Henan Police College, Zhengzhou 450046, China

**Keywords:** LEO satellites, signals of opportunity, inertial navigation system, adaptive unscented Kalman filter, integrated navigation

## Abstract

With the rapid development of low earth orbit (LEO) constellations, LEO signals of opportunity (LEO-SOP) navigation has attracted extensive attention for positioning services in complex-denied environments. However, LEO-SOP suffers from limited coverage multiplicity and time-varying observation noise, leading to degraded positioning accuracy and availability. To address this issue, this paper applies an innovation-based adaptive unscented Kalman filtering (AUKF) scheme to LEO/inertial navigation system (INS) tightly coupled integration. By constructing the innovation sequence, the impact mechanism of noise uncertainty on filtering performance is analyzed, and an online noise covariance estimation strategy is designed to achieve dynamic adaptive compensation for both process and measurement noise. Simulation results demonstrate that the proposed method effectively suppresses the destabilizing effects of LEO observation noise and significantly improves the robustness and estimation accuracy of the filter in complex environments.

## 1. Introduction

LEO satellites exhibit distinctive advantages such as low signal attenuation and rapid geometric variation, making them a highly promising alternative navigation source in global navigation satellite system (GNSS)-denied environments [1]. Positioning techniques based on LEO signals of opportunity (LEO-SOP) typically perform receiver position estimation by extracting observables such as Doppler frequency shifts and incorporating known satellite orbital information. However, limited by the insufficient coverage redundancy of existing LEO constellations and the non-navigation-oriented signal framework, positioning technology relying solely on LEO opportunistic signals suffers from inherent deficiencies in positioning accuracy and service availability [2]. To address this limitation, the integration of LEO and inertial navigation systems (INS) can significantly enhance comprehensive navigation performance.

LEO-SOP positioning technologies based on Iridium, Orbcomm, Globalstar, Starlink, and other constellations have been investigated [3,4,5]. The Doppler measurement information is extracted through LEO constellation simulation or real satellite signal collection, and the position estimation of static targets is subsequently realized by adopting the extended Kalman filter or weighted nonlinear least-squares estimator, with positioning accuracy ranging from tens to hundreds of meters. A previous study [6] explores the temporal and spatial variation characteristics of the geometric dilution of precision (GDOP) of the Starlink constellation and analyzes its corresponding positioning performance. To address the problem of insufficient visible satellites in positioning of the single Iridium constellation, a combined positioning method integrating Iridium and Orbcomm constellations has been proposed, which achieves a positioning accuracy better than 150 m [7]. In [8], a multi-constellation fusion architecture with OneWeb, Starlink, Iridium, and Orbcomm for LEO-SOP is proposed, which greatly improves the overall positioning accuracy.

To enable dynamic target positioning, INS have been integrated with LEO-SOP for combined navigation. In [9], an Iridium/INS integrated positioning technique is proposed to achieve dynamic vessel positioning in GNSS-denied environments. A tightly coupled square-root information filter (SRIF) method is developed in [10], which fuses carrier Doppler shift from LEO constellations with INS data to enable navigation with fewer than eight visible satellites. A dynamic initialization method for LEO/INS integrated positioning is presented in [11], which achieves an accuracy on the order of hundreds of meters and eliminates the requirement for static initial alignment in conventional INS/LEO integrated navigation systems. To improve positioning accuracy in GNSS-denied environments, Jardak and Jault [12] proposed a tightly coupled EKF integrating IMU, magnetometers, and LEO Doppler measurements, which significantly reduces navigation errors when precise satellite orbits are available. An Orbcomm satellite tracking framework is constructed in [13], which effectively mitigates the impact of orbital errors through Doppler-aided INS and improves the continuity of dynamic positioning.

To address the inherent limitation of poor geometric configuration in single-constellation systems, multi-constellation LEO/INS integrated positioning methods are further explored. The Iridium/Orbcomm/INS integrated positioning methods are separately implemented in [14,15], both achieving comparable accuracies on the order of hundreds of meters. The feasibility and performance bounds of LEO-SOP/INS integrated navigation using Iridium, Orbcomm, and Globalstar are further validated in [16]. The performance of tightly coupled INS aiding with Orbcomm LEO satellite Doppler measurements is experimentally evaluated in [17], where the proposed framework reduces the final position error from 31.7 m to 8.8 m after 30 s of GNSS unavailability. Collectively, these studies demonstrate that the integration of INS effectively compensates for the geometric deficiencies caused by insufficient visible LEO satellites, making it a fundamental technology for highly reliable dynamic positioning.

In terms of nonlinear state estimation, the standard unscented Kalman filter (UKF) and its adaptive variants have been extensively studied to address unknown or time-varying noise statistics in complex navigation environments. Recently, adaptive UKF schemes have become a well-established and widely applied framework in integrated positioning systems. For instance, Wei et al. applied a Gaussian-AUKF approach to UWB/IMU fusion localization to mitigate multipath interference [18]. Similarly, Jiang et al. integrated innovation-based AUKF models to suppress measurement errors and inaccurate process noise in mixed indoor environments [19]. Furthermore, AUKF combined with self-correcting mechanisms has been successfully implemented for multi-sensor fusion positioning of maglev trains [20]. 

To address the time-varying measurement error problem of LEO satellites, an innovation-based AUKF scheme is introduced and applied to the LEO/INS integrated navigation system in this paper. First, the dynamic model of INS and the Doppler observation model of LEO satellites are established. Subsequently, a noise adaptive adjustment mechanism based on the innovation sequence is introduced to achieve real-time estimation and dynamic updating of both system noise and measurement noise, thereby effectively suppressing error accumulation and mitigating the impact of abrupt noise. The effectiveness of the proposed algorithm is verified through extensive numerical simulations. It should be noted that in the context of this paper, the term ‘robustness’ does not strictly refer to traditional robust filtering against non-Gaussian outliers or unmodeled dynamics. Instead, it specifically denotes the proposed system’s engineering resilience and stable positioning performance when subjected to the time-varying statistical properties of the LEO Doppler measurement noise and frequent satellite topology changes.

The remainder of this paper is organized as follows. Section 2 describes the system model. The proposed adaptive unscented Kalman filter positioning method is presented in Section 3. The correctness and effectiveness of the method are validated via comprehensive simulation experiments in Section 4. Finally, Section 5 concludes the paper with a summary of the main contributions and prospects for future research directions.

## 2. System Dynamics and Measurement Models

The dynamic model and observation model of the LEO/INS integrated system are introduced in this section. The navigation coordinate frame is defined as the East–North–Up (ENU) coordinate system. It should be noted that the pseudorange rate and Doppler frequency are interconvertible for LEO satellites. Therefore, the pseudorange rate and Doppler frequency shift are collectively referred to as Doppler in the following discussion.

### 2.1. Dynamic and INS Error Model

The fundamental principles of INS are well established in the literature and widely utilized in integrated navigation systems [9,10,11]. To estimate INS sensor drifts and receiver clock parameters simultaneously in the tightly coupled integration, the INS error state vector $x_{ins}\in {\mathbb{R}}^{15}$ is defined as:

(1) $$x_{ins}={\left[\begin{array}{ccccc} \delta \phi & \delta v^{n} & \delta p^{n} & b_{g} & b_{a} \end{array}\right]}^{T}$$

where δ(⋅) denotes the error term; ϕ, $v^{n}$, $p^{n}$ denote the three-dimensional attitude, velocity, and position of the receiver, respectively; and $b_{g}$ and $b_{a}$ denote the three-dimensional drift errors of the gyroscope and accelerometer, respectively. 

The INS error dynamic model is as follows:

(2) $$\left\{\begin{array}{c} \delta \dot{\phi }=\delta \phi \times (\delta \omega_{ie}^{n}+\delta \omega_{en}^{n})+(\delta \omega_{ie}^{n}+\delta \omega_{en}^{n})-C_{b}^{n}\delta \omega_{ib}^{b}\\ \delta {\dot{v}}^{n}=f_{sf}^{n}\times \delta \phi +v^{n}\times (2\delta \omega_{ie}^{n}+\delta \omega_{en}^{n})-(2\omega_{ie}^{n}+\omega_{en}^{n})\times \delta v^{n}+C_{b}^{n}b_{a}+C_{b}^{n}b_{g}\\ \delta {\dot{p}}^{n}=\delta v^{n}\\ {\dot{b}}_{g}=-\tau_{g}^{-1}b_{g}+\eta_{g}\\ {\dot{b}}_{a}=-\tau_{a}^{-1}b_{a}+\eta_{a} \end{array}\right.$$

where $f_{sf}^{n}$ denotes the specific force, $\omega_{ie}^{n}$ denotes the Earth’s rotational angular velocity, $\omega_{en}^{n}$ is the angular velocity of the ENU coordinate system relative to the Earth coordinate system, $C_{b}^{n}$ represents the transformation matrix from the body coordinate system to the navigation coordinate system, $\eta_{a}$ and $\eta_{g}$ are the measured white noise of the accelerometer and gyroscope, respectively, and $\tau_{a}$ and $\tau_{g}$ represent the time constants associated with accelerometer and gyroscope drift errors, respectively.

The continuous-time INS error dynamic model can be further shown in matrix form as:

(3) $${\dot{x}}_{INS}=F_{INS}x_{ins}+\omega_{INS}$$

The continuous-time linear system matrix $F_{INS}\in {\mathbb{R}}^{15\times 15}$ is obtained by taking partial derivatives and linearizing the continuous nonlinear INS navigation error differential equations in (2) with respect to the error state vector $x_{ins}$, and the INS process noise $\omega_{INS}$ is modeled as a zero-mean Gaussian random variable. 

In the tightly coupled LEO/INS integrated navigation system, receiver clock bias δt and clock drift δf must be jointly estimated alongside INS states. The clock error dynamics are modeled as a standard two-state random walk process:

(4) $$\left\{\begin{array}{l} c\delta \dot{t}=c\delta f+w_{clkb}\\ c\delta \dot{f}=w_{clkd} \end{array}\right.$$

where c is the speed of light, and $w_{clkb}$, $w_{clkd}$ are zero-mean Gaussian white noises representing the receiver phase noise and frequency drift noise, respectively.

Combining the 15-state INS error vector with the 2-state receiver clock parameters, the complete system dynamic error state vector $x\in {\mathbb{R}}^{17}$ is explicitly defined as:

(5) $$x={\left[x_{ins}^{T},\;\delta t_{u},\;\delta {\dot{t}}_{u}\right]}^{T}$$

where $\delta t_{u}=c\delta t$ and $\delta {\dot{t}}_{u}=c\delta f$ denote the distance-equivalent receiver clock bias error and clock drift error, respectively.

The continuous-time augmented error dynamic equation is formulated as:

(6) $$\dot{x}=Fx+w$$

where $w={\left[w_{INS}^{T},w_{clkb},w_{clkd}\right]}^{T}\in {\mathbb{R}}^{17}$ is the augmented process noise vector, and the continuous-time system matrix $F\in {\mathbb{R}}^{17\times 17}$ is configured as:

(7) $$\begin{array}{c} F=\left[\begin{array}{cc} F_{INS} & 0_{15\times 2}\\ 0_{2\times 15} & F_{clk} \end{array}\right],\;F_{clk}=\left[\begin{array}{cc} 0 & 1\\ 0 & 0 \end{array}\right] \end{array}$$

The discretized form of (6) is as follows:

(8) $$\begin{array}{c} x_{k}=\Phi_{k,k-1}x_{k-1}+W_{k-1} \end{array}$$

where $x_{k}\in {\mathbb{R}}^{17}$ denotes the discretized state error vector at the k-th time step; $\Phi_{k,k-1}=\exp(F\Delta t)\in {\mathbb{R}}^{17\times 17}$ is the discrete state-transition matrix computed over sampling time interval Δt; and $W_{k-1}\in {\mathbb{R}}^{17}$ represents the discretized system process noise vector modeled as a zero-mean Gaussian random variable with covariance matrix $Q_{k-1}$.

### 2.2. LEO Measurement Model

In the LEO navigation system, the instantaneous Doppler frequency shift measured by the receiver is denoted by $d_{true}$ [16]. Meanwhile, the predicted Doppler value $d_{est}$ is computed by incorporating the receiver position and velocity provided by the INS with the ephemeris-derived position and velocity of the LEO satellite. The difference between the observed instantaneous Doppler and the predicted Doppler is adopted as the system measurement, which can be utilized for state estimation and filtering. Furthermore, based on the generalized pseudorange measurement model, the corresponding generalized Doppler measurement model can be derived as follows:

(9) $$\left\{\begin{array}{c} \delta \rho_{LEO}=-e_{LEO}\cdot \delta p^{n}+c\delta t+\varepsilon_{LEO}\\ e_{LEO}=\frac{p_{LEO}^{n}-p^{n}}{\left\|p_{LEO}^{n}-p^{n}\right\|} \end{array}\right.$$

where $\delta \rho_{LEO}$ is the pseudorange residual, $p_{LEO}^{n}$ denotes the position vector of the LEO satellite, $\varepsilon_{LEO}$ is the observed pseudorange noise, which is modeled as a zero-mean Gaussian random variable, and $e_{LEO}\in {\mathbb{R}}^{3}$ denotes the 3D line-of-sight (LOS) unit vector pointing from the receiver position to the LEO satellite position.

The generalized Doppler measurement model is obtained through differentiation of (9):

(10) $$\left\{\begin{array}{c} \delta {\dot{\rho }}_{LEO}=-{\dot{e}}_{LEO}\cdot \delta p^{n}-e_{LEO}\cdot \delta v^{n}+c\delta f+{\dot{\varepsilon }}_{LEO}\\ {\dot{e}}_{LEO}=\frac{(v_{LEO}^{n}-v^{n})-e_{LEO}\cdot \left[e_{LEO}\cdot (v_{LEO}^{n}-v^{n})\right]}{\left\|p_{LEO}^{n}-p^{n}\right\|} \end{array}\right.$$

where ${\dot{\varepsilon }}_{LEO}$ represents the observed Doppler noise, modeled as a zero-mean Gaussian random variable with variance R.

The relationship between the measured Doppler frequency shift $f_{d}^{\left(i\right)}$ and the pseudorange rate ${\dot{\rho }}^{\left(i\right)}$ for the i-th visible LEO satellite is expressed as:

(11) $${\dot{\rho }}^{\left(i\right)}=-\lambda \cdot f_{d}^{\left(i\right)}=-\frac{c}{f_{0}}\cdot f_{d}^{\left(i\right)}$$

where $f_{0}$ denotes the nominal carrier frequency of the LEO transmission signal, and $\lambda =c/f_{0}$ is the carrier wavelength.

## 3. The Proposed Method

LEO/INS integrated navigation is a typical nonlinear estimation problem requiring nonlinear filtering. To mitigate time-varying measurement noise caused by multipath and other interference in LEO signals, an adaptive unscented Kalman filtering framework is employed. In this work, equivalent noise statistics are estimated in real time from the innovation sequence accumulated over a sliding window, and the process noise covariance and measurement noise covariance are subsequently adjusted to improve the state estimation.

### 3.1. Adaptive Unscented Kalman Filter

The UKF has become a cornerstone in nonlinear estimation and has been widely successfully applied in integrated navigation systems [18,20]. Unlike the EKF, which relies on first-order Taylor expansion, the UKF captures the posterior mean and covariance accurately to the third order for Gaussian inputs via the unscented transformation (UT).

The discrete-time LEO/INS integrated navigation system model may be simplified as

(12) $$\left\{\begin{array}{l} x_{k}=\Phi_{k,k-1}x_{k-1}+w_{k-1}\\ z_{k}=h(x_{k})+v_{k} \end{array}\right.$$

where $\omega_{k-1}$ and $v_{k}$ are the zero-mean Gaussian process noise and measurement noise, respectively, with corresponding covariance matrices $Q_{k}$ and $R_{k}$.

The posterior probability density function of the system is assumed as a Gaussian distribution, expressed as:

(13) $$p(x_{k-1}|z_{1:k-1})=N(x_{k-1};{\hat{x}}_{k-1},P_{k-1})$$

where $N(x_{k-1};{\hat{x}}_{k-1},P_{k-1})$ denotes the Gaussian probability density function of the state error vector $x_{k-1}\in {\mathbb{R}}^{17}$ parameterized by mean vector ${\hat{x}}_{k-1}\in {\mathbb{R}}^{17}$ and error covariance matrix $P_{k-1}\in {\mathbb{R}}^{17\times 17}$; and $z_{1:k-1}$ represents the accumulated measurement sequence up to epoch k−1.

The covariance matrix is decomposed via Cholesky factorization as

(14) $$P_{k-1}=S_{k-1}S_{k-1}^{T}$$

where $S_{k-1|k-1}$ is a lower triangular matrix.

To incorporate process noise and additional variables into the filtering recursion, the state vector is first augmented to dimension *n*, then a set of 2*n* + 1 sigma points is deterministically generated from the augmented posterior distribution to parameterize the unscented transformation.

(15) $$\chi_{k-1}^{\left(i\right)}=\left\{\begin{array}{l} {\hat{x}}_{k-1},\;\;\;\;\;\;\;\;i=0\\ {\hat{x}}_{k-1}+{\left(\sqrt{(n+\lambda )P_{k-1}}\right)}_{i},\;i=1,\ldots ,n\\ {\hat{x}}_{k-1}-{\left(\sqrt{(n+\lambda )P_{k-1}}\right)}_{i-n},\;i=n+1,\ldots ,2n \end{array}\right.$$

where n is the dimension of the state vector, λ is the scaling parameter, and ${\left(\sqrt{P}\right)}_{i}$ denotes the i-th column of the lower triangular matrix obtained from the Cholesky decomposition of P. 

The associated weights for the mean and covariance are determined by the scaling parameter λ, which is defined as

(16) $$\lambda =\alpha^{2}(n+\kappa )-n$$

where α and κ control the spread and scaling of the sigma points, respectively.

The sigma-point weights are prescribed by the standard unscented transform in terms of λ, where the center-point mean and covariance weights are respectively defined as:

(17) $$\left\{\begin{array}{l} \omega_{0}^{\left(m\right)}=\frac{\lambda }{n+\lambda }\\ \omega_{0}^{\left(c\right)}=\frac{\lambda }{n+\lambda }+(1-\alpha^{2}+\beta ) \end{array}\right.$$

where β is the prior distribution parameter.

The remaining sigma points have the same weights:

(18) $$\omega_{i}^{\left(m\right)}=\omega_{i}^{\left(c\right)}=\frac{1}{2(n+\lambda )},\;i=1,2,\ldots ,2n$$

The sigma points generated at the previous time step are propagated through the nonlinear state dynamics to yield the corresponding predicted points for the time update.

(19) $$\chi_{i,k|k-1}=f(\chi_{i,k-1})$$

The predicted state mean at time *k* is subsequently computed from the propagated sigma points as

(20) $${\hat{x}}_{k|k-1}=\sum_{i=0}^{2n}\omega_{i}^{\left(m\right)}\chi_{i,k|k-1}$$

The predicted state covariance is subsequently estimated as:

(21) $$P_{k|k-1}=\sum_{i=0}^{2n}\omega_{i}^{\left(c\right)}(\chi_{i,k|k-1}-{\hat{x}}_{k|k-1}){\left(\chi_{i,k|k-1}-{\hat{x}}_{k|k-1}\right)}^{T}+Q_{k-1}$$

The predicted sigma points are then passed through the nonlinear measurement model to yield:

(22) $$Z_{i,k|k-1}=h(\chi_{i,k|k-1})$$

The predicted measurement mean is given by:

(23) $${\hat{z}}_{k|k-1}=\sum_{i=0}^{2n}\omega_{i}^{\left(m\right)}Z_{i,k|k-1}$$

Based on the distribution of the propagated sigma points in the measurement space, the measurement covariance is obtained as:

(24) $$P_{zz,k|k-1}=\sum_{i=0}^{2n}\omega_{i}^{\left(c\right)}(Z_{i,k|k-1}-{\hat{z}}_{k|k-1}){\left(Z_{i,k|k-1}-{\hat{z}}_{k|k-1}\right)}^{T}+R_{k}$$

The cross-covariance between the state and the measurement is computed as:

(25) $$P_{xz,k|k-1}=\sum_{i=0}^{2n}\omega_{i}^{\left(c\right)}(\chi_{i,k|k-1}-{\hat{x}}_{k|k-1}){\left(Z_{i,k|k-1}-{\hat{z}}_{k|k-1}\right)}^{T}$$

The Kalman gain is then obtained as:

(26) $$K_{k}=P_{xz,k|k-1}{P_{zz,k|k-1}}^{-1}$$

The state estimate is updated as:

(27) $${\hat{x}}_{k}={\hat{x}}_{k|k-1}+K_{k}(z_{k}-{\hat{z}}_{k|k-1})$$

The corresponding covariance matrix is updated as:

(28) $$P_{k}=P_{k|k-1}-K_{k}P_{zz,k|k-1}K_{k}^{T}$$

To enhance the adaptivity and resilience of the filter against unknown or time-varying noise statistics, an online covariance matching mechanism based on the innovation sequence is constructed. The innovation sequence $v_{k}$ is defined as the difference between the true measurement and the predicted measurement:

(29) $$v_{k}=z_{k}-{\hat{z}}_{k|k-1}$$

To dynamically estimate the unknown noise statistics, the innovation covariance is continuously approximated over a moving window of length N. The statistically estimated innovation covariance ${\hat{C}}_{v,k}$ at time step k is given by:

(30) $${\hat{C}}_{v,k}=\frac{1}{N}\sum_{j=0}^{N-1}v_{k-j}v_{k-j}^{T}$$

According to the classical covariance matching estimation strategy [21,22,23], the theoretical innovation covariance is intrinsically composed of the propagated state uncertainty and the measurement noise. By matching the theoretical covariance with the statistically estimated ${\hat{C}}_{v,k}$, the adaptive measurement noise covariance ${\hat{R}}_{k}$ can be isolated and dynamically updated:

(31) $${\hat{R}}_{k}={\hat{C}}_{v,k}-\sum_{i=0}^{2n}W_{i}^{\left(c\right)}(Z_{i,k|k-1}-{\hat{z}}_{k|k-1}){\left(Z_{i,k|k-1}-{\hat{z}}_{k|k-1}\right)}^{T}$$

Unlike the measurement noise, accurately estimating the process noise covariance requires projecting the innovation information back into the state space. Utilizing the orthogonality principle between the innovation sequence and the state estimation error, the adaptive process noise covariance ${\hat{Q}}_{k}$ can be iteratively estimated via the Kalman gain $K_{k}$:

(32) $${\hat{Q}}_{k}=K_{k}{\hat{C}}_{v,k}K_{k}^{T}+P_{k|k}-\sum_{i=0}^{2n}W_{i}^{\left(c\right)}(\chi_{i,k|k-1}-{\hat{x}}_{k|k-1}){\left(\chi_{i,k|k-1}-{\hat{x}}_{k|k-1}\right)}^{T}$$

To prevent potential violations of the nonnegative definiteness condition and to ensure numerical stability during the recursion, a complete covariance regularization procedure is implemented. First, the statistically estimated matrices are explicitly symmetrized via ${\hat{Q}}_{k}=({\hat{Q}}_{k}+{\hat{Q}}_{k}^{T})/2$ and ${\hat{R}}_{k}=({\hat{R}}_{k}+{\hat{R}}_{k}^{T})/2$. Second, to reduce the computational burden and prevent filter divergence caused by spurious cross-correlations, the estimated covariance matrices are strictly constrained to be diagonal matrices. Finally, the diagonal elements are constrained by predefined positive lower bounds. If any diagonal element ${\hat{Q}}_{k}(i,i)\;or\;{\hat{R}}_{k}(j,j)$ falls below the physical threshold $q_{min}\;or\;r_{min}$, it is forced to equal the lower bound. In our implementation, the moving-window length is set to N=20. The lower bounds are set proportionally to the baseline noise parameters as $q_{min}=10^{-4}\cdot Q_{0}(i,i)$ and $r_{min}=10^{-2}\cdot R_{0}(j,j)$, ensuring physical validity without suppressing necessary filter adaptation.

Simultaneously estimating the process noise covariance Q and measurement noise covariance R from the same innovation sequence intrinsically risks identifiability and numerical-stability problems, as the innovation covariance mathematically represents the coupled uncertainty of both dynamics and measurements. To resolve this, our algorithm avoids unconstrained simultaneous estimation. The adaptation is guided by the distinct dynamic characteristics of the LEO/INS integration: the measurement noise covariance R responds rapidly to high-frequency LEO observation anomalies, whereas the process noise covariance Q is restricted to slower, bounded variations reflecting INS model drift. By anchoring the estimations with strict diagonal constraints and physical lower bounds derived from IMU specifications, the identifiability of the noise parameters is preserved. Transient LEO signal anomalies are thereby forced to scale ${\hat{R}}_{k}$ exclusively, rather than indiscriminately inflating ${\hat{Q}}_{k}$.

In non-stationary time-varying integrated systems, strict asymptotic convergence proofs for joint state and noise estimation are mathematically intractable due to switching topologies. Instead, the proposed diagonal lower-bound regularization helps preserve positive definiteness and improves numerical stability by preventing covariance collapse and matrix singularities during the filtering recursion. By maintaining strictly positive-definite process and measurement noise covariances, this regularization effectively prevents numerical degradation and keeps the Kalman gain and posterior error covariance within stable operational bounds, thereby mitigating the risk of filter divergence under transient observation anomalies.

### 3.2. LEO/INS Integrated Positioning

**Figure 1:**
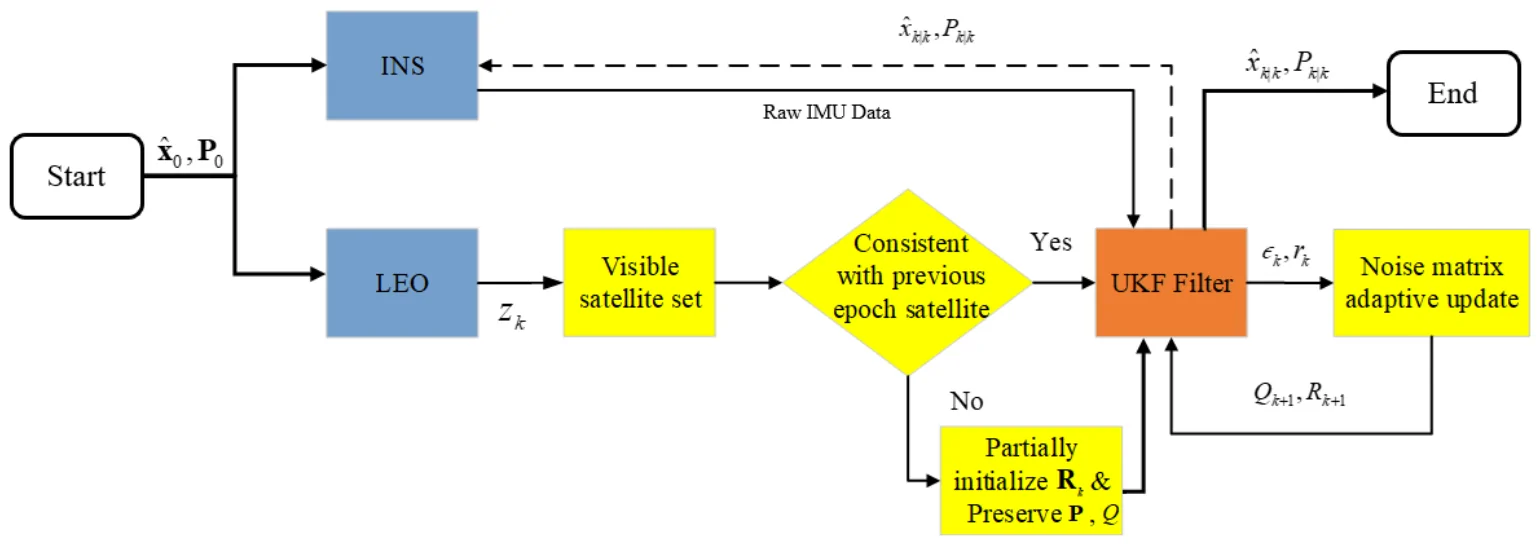
The proposed LEO/INS integrated positioning algorithm.

The high orbital velocity and short visibility of LEO satellites cause frequent changes in the visible satellite set during LEO/INS integrated positioning, leading to time-varying observation geometry and measurement dimensionality. The flowchart of the proposed LEO/INS integrated positioning algorithm is shown in Figure 1. The filter initializes at the Start node with initial state estimate ${\overset{ˆ}{x}}_{0}$ and error covariance $P_{0}$. At each epoch k, raw IMU measurements are fed from the INS to unconditionally execute state prediction via (20) and (21), propagating predicted mean ${\overset{\wedge }{x}}_{k|k-1}$ and error covariance $P_{k|k-1}$. Subsequently, the visible LEO satellite set is recorded and compared across consecutive epochs. When the satellite set remains unchanged, the observation model is structurally stable, and process and measurement noise covariances $Q_{k}$ and $R_{k}$ are updated via the proposed adaptive mechanism. Conversely, when a satellite configuration change is detected, the state error covariance $P_{k|k-1}$ is strictly preserved and propagated to retain accumulated estimation memory of INS sensor biases and clock parameters. When a satellite configuration change is detected, the state error covariance $P_{k|k-1}$ and the process noise covariance $Q_{k}$ are strictly preserved and propagated. This is because $Q_{k}$ is exclusively determined by the internal INS dynamics, and $P_{k|k-1}$ retains the continuous state estimation memory, neither of which is affected by external LEO satellite topology changes. For the measurement noise covariance $R_{k}$, it is dynamically resized rather than fully reinitialized. Specifically, if a previously tracked satellite becomes invisible, its corresponding row and column in $R_{k}$ are directly deleted; if a new satellite is acquired, only the newly added satellite’s measurement variance is initialized and appended to $R_{k}$. This strategy ensures that the valuable accumulated covariance information for the continuously tracked satellites is strictly preserved without contamination.

The proposed adaptive UKF for LEO/INS integrated navigation is summarized in Algorithm 1.
**Algorithm 1.** The proposed LEO/INS integrated positioning algorithmInput: Visible LEO satellite positions and velocities, raw INS IMU measurements, initial state estimate ${\overset{ˆ}{x}}_{0}$, initial covariance $P_{0}$, and nominal noise matrices.Output: Calibrated navigation state estimate ${\overset{ˆ}{x}}_{k}$ and posterior error covariance matrix $P_{k}$ at each discrete epoch k.1: Obtain LEO Doppler shift measurements from LEO satellites. 2: Propagate INS state dynamics using raw IMU data and unconditionally compute predicted state mean ${\hat{x}}_{k|k-1}$ and predicted error covariance $P_{k|k-1}$ via (20) and (21). 3: **If** the set of visible LEO satellites differs from that of the previous epoch **then** 4: Preserve $P_{k|k-1}$ and $Q_{k}$. Dynamically resize $R_{k}$ by deleting the variance elements of disappeared satellites and initializing only the variance elements for newly acquired satellites. 5: **Else** 6: Adaptively update $Q_{k}$ and $R_{k}$ via (31) and (32). 7: **End if** 8: Compute the predicted measurement mean, innovation covariance, and cross-covariance via (23), (24), and (25), respectively. 9: Compute the Kalman gain via (26). 10: Update the state estimate and covariance via (27) and (28), respectively.11: Output ${\overset{ˆ}{x}}_{k}$ and $P_{k}$, calibrate INS errors, and proceed to epoch k+1 and return to Step 1.

When the visible LEO satellite set changes, the observation mapping matrix $H_{k}$ undergoes a dimensional transition. Instead of completely flushing the sliding window and fully resetting $R_{k}$, which would repeatedly discard valuable accumulated covariance information, $R_{k}$ is partially initialized. Specifically, the variance elements corresponding to disappeared satellites are deleted, and only the variance elements for newly acquired satellites are initialized to their nominal value $R_{0}$. Furthermore, the process noise covariance $Q_{k}$ is exclusively determined by the INS dynamics, and the predicted error covariance $P_{k|k-1}$ resides in the continuous state space. Both are intrinsically invariant to external LEO satellite topology changes. Therefore, preserving $P_{k|k-1}$ and $Q_{k}$ continuously retains the accumulated INS bias calibration memory and process noise characteristics, ensuring smooth state transitions without filter re-warmup.

## 4. Simulation Results

The performance of the proposed AUKF for LEO/INS integrated navigation is evaluated through a vehicle-based simulation over a total duration of 272 s. The simulation adopts the ENU navigation frame, with an initial velocity of zero, an INS update rate of 100 Hz, and LEO measurement updates of 1 Hz. Orbital trajectories of the LEO satellites were generated using STK 12.4 software based on actual two-line element (TLE) data files. An elevation mask of $10^{{}^{\circ}}$ was applied, yielding a sparse visible satellite count dynamically varying between 1 and 3. LEO Doppler measurements were synthesized assuming a nominal carrier frequency of $f_{0}=1.626\;\mathrm{GHz}$ by projecting relative satellite-receiver velocity vectors along the line of sight, superimposed with receiver clock drift, a 100 m 3D RMS orbit error, and a zero-mean 1 Hz Gaussian measurement noise. The UKF scaling parameters were configured to standard values: $\alpha =10^{-3}$, β=2, and κ=0. Two distinct error levels are considered to comprehensively assess the algorithm, along with configurations both with and without altimeter aiding, in order to examine the impact of vertical aiding information on the integrated positioning accuracy.

It should be clarified that in the context of LEO-SOP navigation, the LEO satellites act as moving transmitters in space, while the navigation target is the dynamic receiver. Therefore, to evaluate the integrated positioning performance, a ground vehicle equipped with an INS and a LEO signal receiver is utilized as the dynamic platform in this simulation.

**Figure 2:**
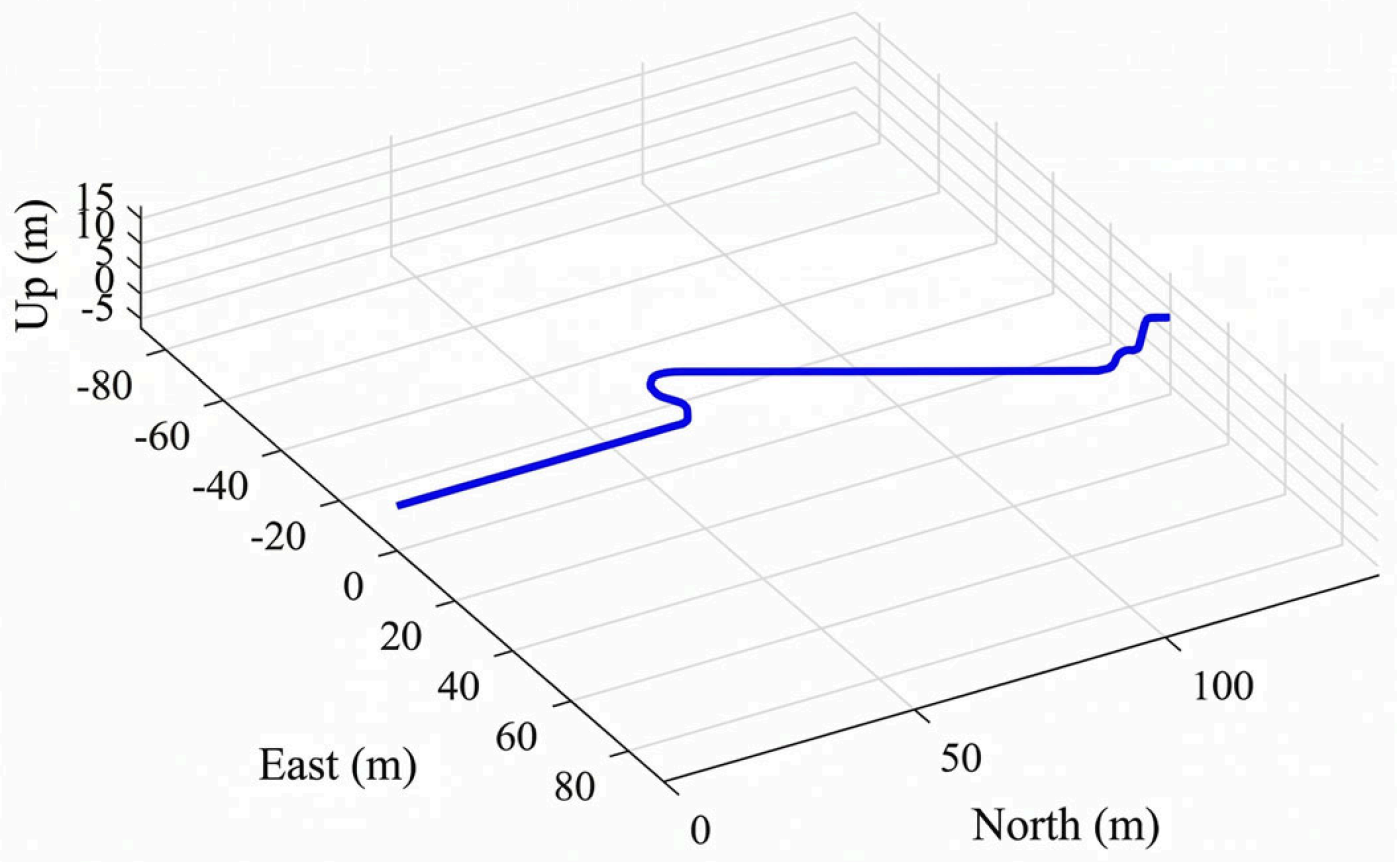
The simulated vehicle trajectory.

The simulated vehicle trajectory is illustrated in Figure 2, which incorporates typical dynamic characteristics of on-road driving scenarios. The motion initiates from rest with a smooth acceleration to approximately 20 m/s, followed by two extended segments of constant-speed cruising. The subsequent portion incorporates a series of turning maneuvers, including 90° left and right turns as well as gentle continuous weaving, superimposed with mild roll dynamics to emulate realistic cornering behavior. A modest slope variation of approximately 2° is further introduced to capture pitch and altitude changes associated with road undulations. The trajectory concludes with a gradual deceleration to a complete stop, accompanied by a slight pitch response. Overall, the motion profile integrates acceleration, cruising, turning, slope transitions, and braking phases. 

In the altimeter-aided configurations, the vertical position is treated as an idealized reference to effectively constrain vertical channel drift and highlight the baseline performance improvement on the horizontal components. It is worth noting that practical altimeter sensor noise and biases were not modeled in this preliminary evaluation. The first simulation scenario evaluates the positioning performance under low sensor noise conditions, with the detailed simulation parameters summarized in Table 1.

To ensure a fair and rigorous comparison among the evaluated algorithms, it is essential to specify the covariance configurations. In the simulations, the standard EKF, the standard UKF, and the proposed AUKF were all initialized with identical initial process noise covariance $Q_{0}$ and measurement noise covariance $R_{0}$. These initial values were chosen empirically based on the nominal sensor specifications. Specifically, the initial state covariance $P_{0}$ and process noise covariance $Q_{0}$ are constructed as diagonal matrices using nominal standard deviations: attitude error $\sigma_{att}=0.5^{{}^{\circ}}$, velocity error $\sigma_{vel}=1\;m/s$, position error $\sigma_{pos}=50\;m$, gyro drift $\sigma_{gyro}=3^{{}^{\circ}}/h$, accelerometer bias $\sigma_{acc}=100\;\mu g$, clock bias $\sigma_{clkb}=100\;m$, and clock drift $\sigma_{clkd}=1\;m/s$. The initial measurement noise covariance is configured as $R_{0}=1.0\;{\mathrm{Hz}}^{2}\cdot I_{m\times m}$.

However, to simulate real-world non-stationary observation conditions, time-varying measurement noise was introduced along the trajectory. Specifically, the true Doppler measurement noise variance remained at the nominal baseline level $R_{true}=1.0\;{\mathrm{Hz}}^{2}$ during steady tracking periods, but surged to 3.0–5.0 ${\mathrm{Hz}}^{2}$ during t = 103 s and t = 172 s. Throughout the simulation, the standard EKF and UKF utilized the fixed nominal covariance $R_{0}=1.0\;{\mathrm{Hz}}^{2}$, which induced intentional mismatches during transient surge periods. In contrast, the proposed AUKF dynamically estimated and compensated for these time-varying noise statistics online via the innovation-based adaptive mechanism, thus achieving superior convergence and robustness.

**Figure 3:**
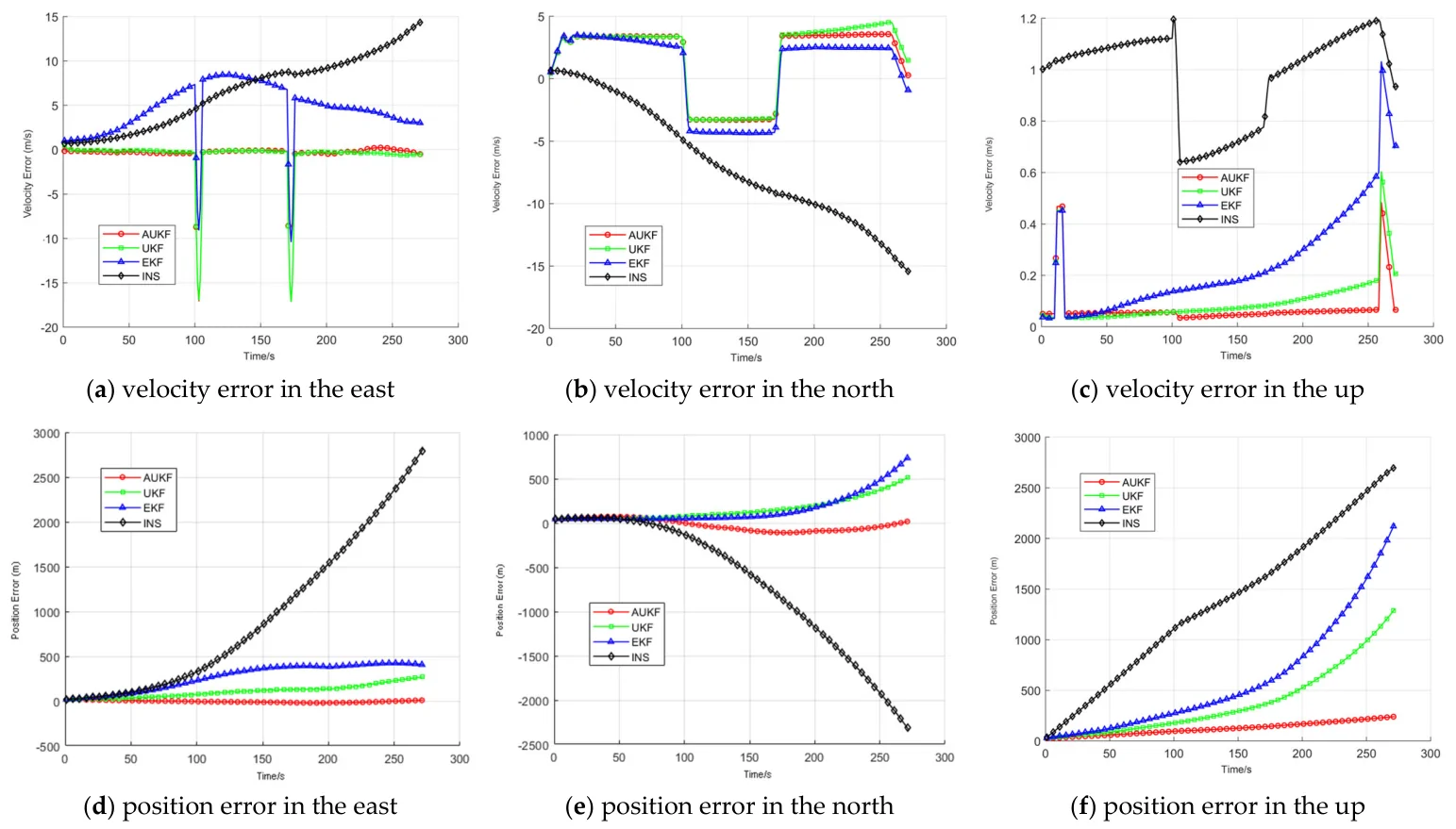
Positioning error comparisons under low-noise conditions without altimeter aiding.

Figure 3 presents the positioning error comparisons among different algorithms under the low-noise conditions without altimeter aiding. The corresponding RMSE comparison is shown in Table 2, where p-e, p-n, p-u, and p-3D denote the position RMSE in the east, north, and up directions and overall, respectively, while v-e, v-n, v-u, and v-3D denote the corresponding velocity RMSE in the east, north, up directions and overall. Figure 3a–c illustrates the velocity errors in the east, north, and up directions. It can be seen that the EKF, UKF, and the proposed AUKF eventually achieve convergence, while the performance differs considerably. The proposed AUKF yields the smoothest velocity profiles with pronounced stability, and the UKF maintains adequate stability during highly nonlinear intervals, whereas the EKF exhibits distinct spikes in the vicinity of satellite switches or measurement discontinuities, indicating a heightened sensitivity to noise uncertainty and observation anomalies. The position errors in the east, north, and up directions are shown in Figure 3d–f. As illustrated in Figure 3, the position error evolves in a highly consistent trend with the velocity error. Benefiting from the geometric distribution of the integrated satellite constellation, the north direction exhibits superior observability. Accordingly, the north position error is less sensitive to measurement noise, leading to a minor performance discrepancy among different algorithms. As shown in Table 2, AUKF achieves reductions in 3-D position RMSE of approximately 49.9% over UKF, 82.3% over EKF, and 93.1% over INS. For velocity RMSE, the corresponding reductions are 5.3%, 39.5%, and 71.7% relative to UKF, EKF, and INS, respectively. These results indicate that the proposed AUKF effectively mitigates the accumulation of system noise, thereby attaining superior fusion accuracy among the evaluated methods.

**Figure 4:**
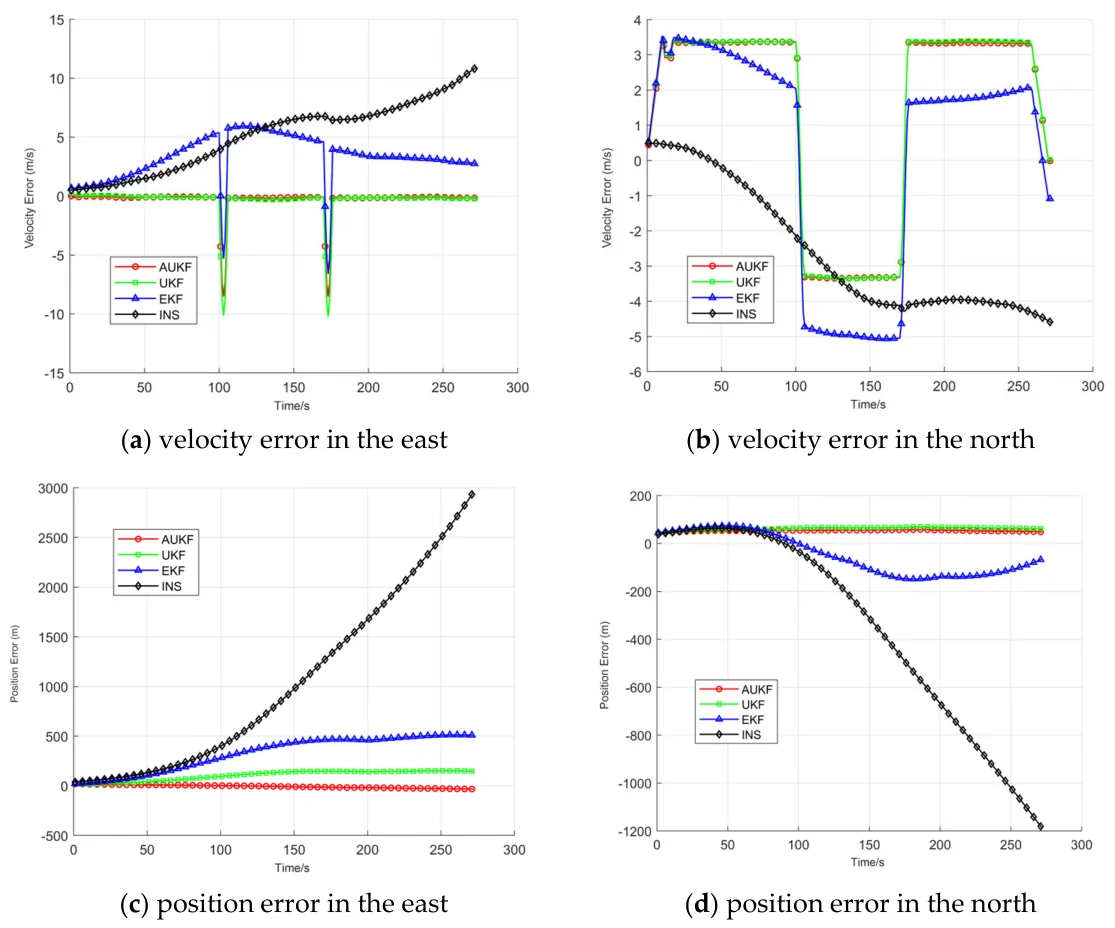
Positioning error comparisons under low-noise conditions with altimeter aiding.

Figure 4 presents the positioning error comparisons among different algorithms under the low-noise conditions with altimeter aiding. The corresponding RMSE comparison is shown in Table 3. It can be seen that both position and velocity errors are substantially reduced for all algorithms under altitude assistance compared to the unassisted case. Among the evaluated methods, the proposed AUKF consistently exhibits the smoothest and most rapid convergence, UKF remains stable throughout, and EKF displays only minor residual oscillations with markedly reduced amplitude at LEO measurement transitions. Quantitatively, the proposed AUKF reduces velocity RMSE by 27.4%, 71.0%, and 78.3% relative to UKF, EKF, and INS, respectively. With respect to horizontal position error, the proposed AUKF maintains enhanced convergence characteristics, whereas EKF still exhibits subtle fluctuations during measurement transitions. Correspondingly, the proposed AUKF achieves reductions in horizontal position RMSE of 23.8% over UKF, 86.9% over EKF, and 95.4% over INS, which is further accentuated relative to the unassisted scenario.

The second simulation scenario evaluates the positioning performance under large sensor noise conditions, with the detailed simulation parameters summarized in Table 4.

**Figure 5:**
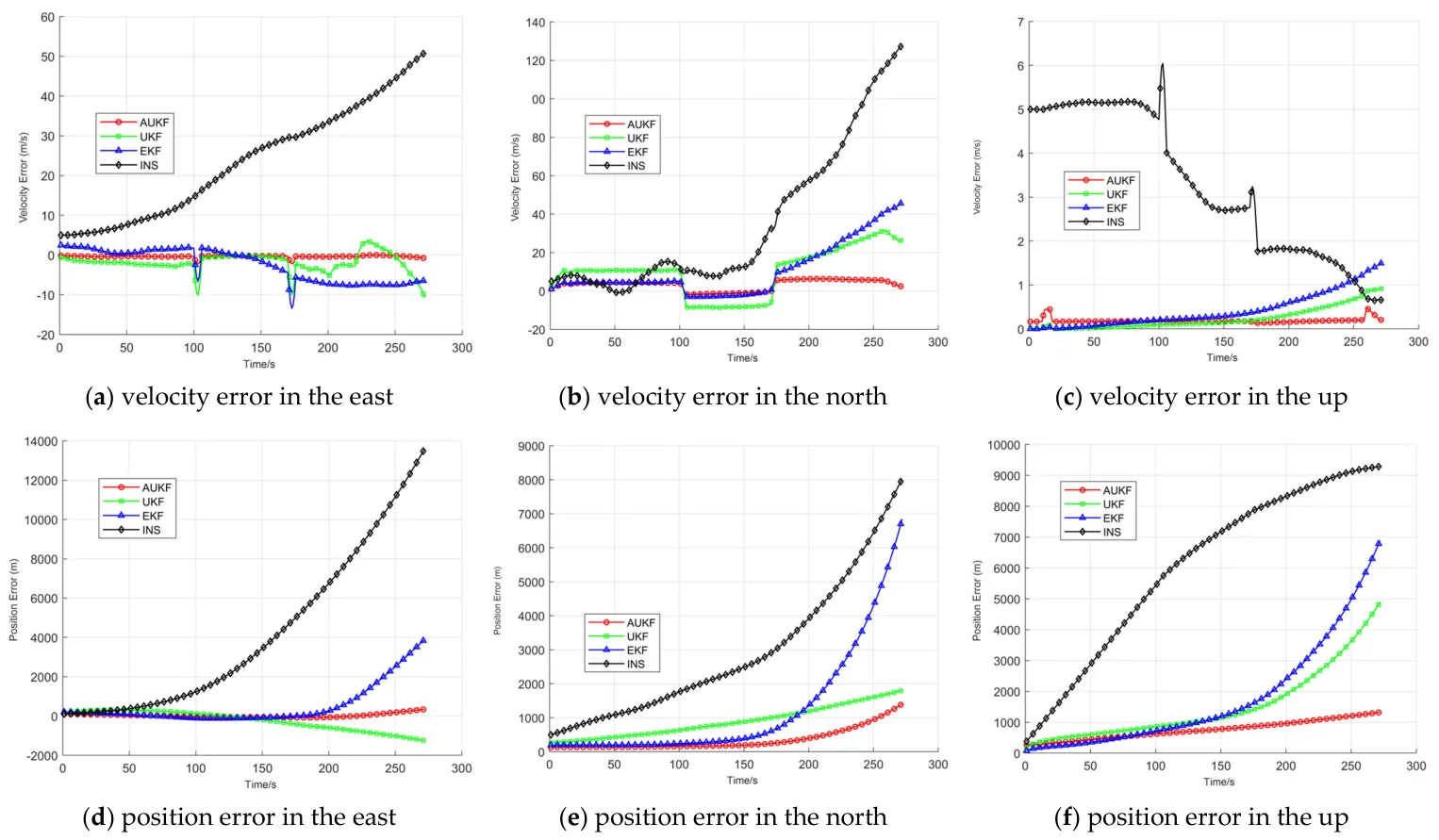
Positioning error comparisons under high-noise conditions without altimeter aiding.

Figure 5 presents the positioning error comparisons among different algorithms under the high-noise conditions without altimeter aiding, and the corresponding RMSE statistics are summarized in Table 5. It can be seen that the large initial errors markedly amplify system sensitivity to noise and model uncertainty. The EKF exhibits pronounced oscillations with quasi-divergent behavior in certain intervals, while UKF maintains acceptable convergence albeit with notably elevated overall error. In contrast, the proposed AUKF preserves the most stable trajectory, achieving reductions in velocity RMSE of 52.4%, 71.7%, and 88.7% relative to UKF, EKF, and INS, respectively. Furthermore, the discrepancy in position error under high-noise conditions is even more pronounced than that in velocity. Notably, during periods of satellite transition, EKF generates substantial spikes, whereas AUKF exhibits comparatively smooth error variation. Correspondingly, the proposed AUKF reduces 3-D position RMSE by 63.8% over UKF, 80.4% over EKF, and 92.8% over INS. 

**Figure 6:**
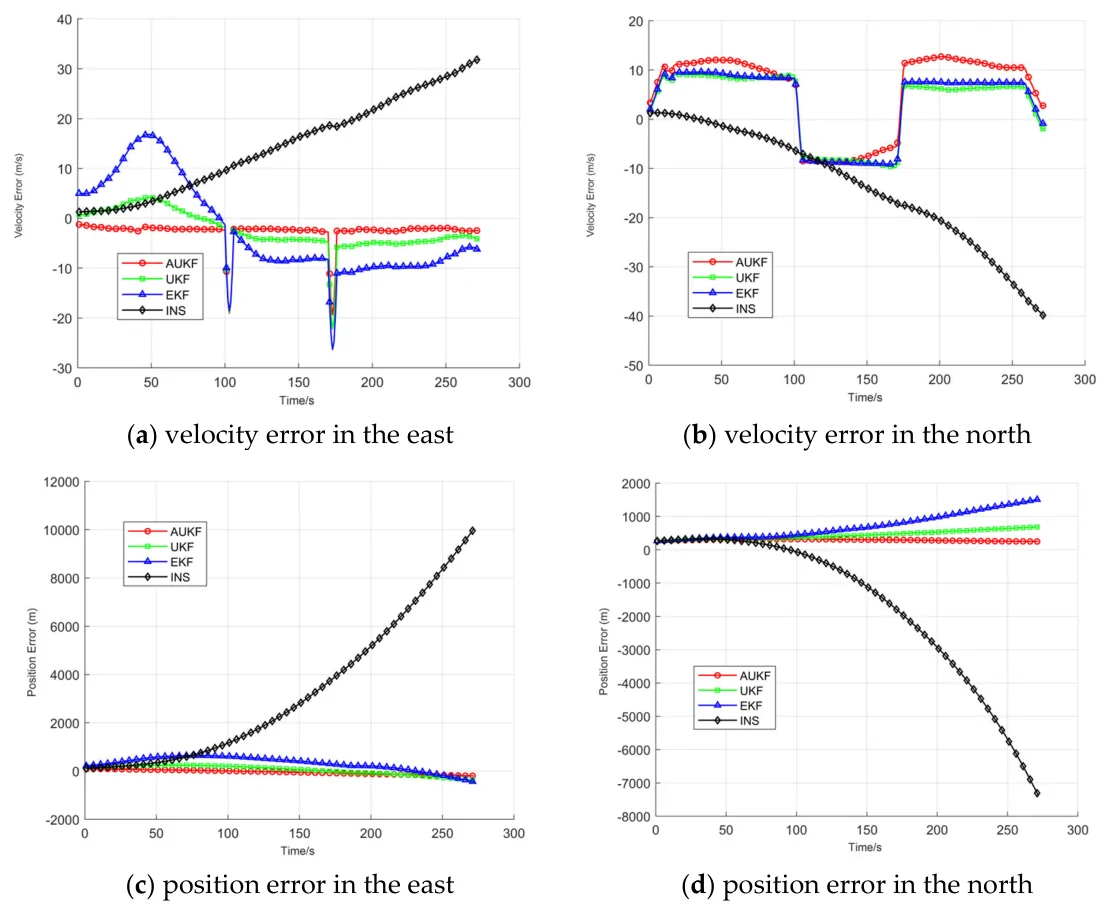
Positioning error comparisons under high-noise conditions with altimeter aiding.

Figure 6 illustrates positioning error comparisons among different algorithms under large-error conditions with altitude assistance, and the corresponding error statistics are summarized in Table 6. The inclusion of altitude assistance effectively mitigates the pronounced performance degradation induced by large initial errors, enabling convergence of all three methods and yielding overall error trends that more closely resemble those observed under small-error conditions. As shown in Table 6, the proposed AUKF achieves reductions in horizontal position RMSE of 36.3% relative to UKF, 68.7% relative to EKF, and 93.9% relative to INS. For velocity RMSE, corresponding reductions of 19.4%, 53.7%, and 64.6% are attained compared to UKF, EKF, and INS, respectively, demonstrating consistently superior overall performance.

## 5. Conclusions

This paper focuses on the fusion demand of LEO-SOP and INS in complex dynamic environments and employs an adaptive unscented Kalman filtering strategy to improve the performance of the integrated navigation. A Doppler-based observation model tailored to LEO satellite characteristics is systematically derived and embedded within the INS feedback correction loop, establishing a rigorous framework for incorporating opportunistic LEO measurements into the filtering update. Aiming at the time-varying measurement error of LEO satellites, a noise adaptive adjustment mechanism based on the innovation sequence is introduced, which realizes real-time estimation and dynamic updating of both system noise and measurement noise, thereby effectively suppressing error accumulation and mitigating the impact of abrupt noise caused by frequent changes of visible satellite combinations. The simulation results demonstrate that the proposed AUKF algorithm exhibits superior resilience and positioning accuracy in various typical scenarios, including low-error and high-error and with and without altitude aiding conditions. It should be noted that these findings are established based on temporal RMSE evaluations over a simulated dynamic trajectory under an idealized vertical constraint simplification, as independent Monte Carlo realizations, comparative benchmarks with other adaptive filtering variants, real-world LEO signal captures, and dynamic vehicle field-test data were not incorporated in this study. To further enhance navigation performance and statistical validation, our future research will conduct comprehensive Monte Carlo analyses, benchmark against established adaptive filtering schemes, integrate realistic altimeter noise models, and explore elevation-dependent weighting mechanisms to dynamically optimize measurement noise covariance estimation.

## Figures and Tables

**Table 1:** Sensor parameters for low-noise conditions.

Parameter	Value
Gyroscope bias	3°/h
Gyroscope random walk	1°/√h
Accelerometer bias	100 μg
Accelerometer random walk	5 μg/√Hz
Initial INS attitude error	[0.5, −0.5, 20]°
Initial INS velocity error	[1, 1, 1] m/s
Initial INS position error	[50, 20, 38] m
Doppler frequency measurement error	1 Hz

**Table 2:** Corresponding RMSE comparison of Figure 3.

Method	p-e (m)	p-n (m)	p-u (m)	p-3D (m)	v-e (m/s)	v-n (m/s)	v-u (m/s)	v-3D (m/s)
AUKF	16.95	51.75	140.63	150.805	0.834	3.542	0.087	3.639
UKF	42.55	110.65	276.45	300.796	0.957	3.721	0.134	3.844
EKF	363.20	201.75	743.61	851.805	4.711	3.724	0.372	6.016
INS	1257.15	916.40	1527.44	2180.203	7.074	10.678	1.052	12.851

**Table 3:** Corresponding RMSE comparison of Figure 4.

Method	p-e (m)	p-n (m)	p-2D (m)	v-e (m/s)	v-n (m/s)	v-2D (m/s)
AUKF	13.45	48.65	50.476	0.259	1.931	1.949
UKF	20.15	63.15	66.280	0.408	2.654	2.685
EKF	349.1	162.6	385.090	5.534	3.835	6.735
INS	968.21	498.12	1088.850	6.221	6.439	8.950

**Table 4:** Sensor parameters for high-noise conditions.

Parameter	Value
Gyroscope bias	10°/h
Gyroscope random walk	5°/√h
Accelerometer bias	500 μg
Accelerometer random walk	50 μg/√Hz
Initial INS attitude error	[5, −5, 50]°
Initial INS velocity error	[5, 5, 5] m/s
Initial INS position error	[500, 200, 380] m
Doppler frequency measurement error	1 Hz

**Table 5:** Corresponding RMSE comparison of Figure 5.

Method	p-e (m)	p-n (m)	p-u (m)	p-3D (m)	v-e (m/s)	v-n (m/s)	v-u (m/s)	v-3D (m/s)
AUKF	84.56	215.36	685.27	720.687	2.734	5.175	0.276	5.871
UKF	275.35	652.37	1854.36	1990.668	4.258	11.571	0.485	12.329
EKF	985.68	2036.25	2845.21	3690.571	6.225	19.758	0.688	20.736
INS	7532.36	3785.58	4982.33	10,055.846	24.521	45.845	3.046	52.183

**Table 6:** Corresponding RMSE comparison of Figure 6.

Method	p-e (m)	p-n (m)	p-2D (m)	v-e (m/s)	v-n (m/s)	v-2D (m/s)
AUKF	175.25	215.36	277.655	1.268	8.687	8.7791
UKF	253.68	354.26	435.722	2.587	10.584	10.8956
EKF	452.58	763.95	887.946	13.587	13.247	18.9760
INS	3459.64	2927.44	4531.998	15.185	19.634	24.8209

## Data Availability

The data presented in this study are available on request from the corresponding author.
